# Lactate induces renal lipid accumulation and aggravates renal fibrosis by inhibiting the PPARα signaling pathway and fatty acid oxidation

**DOI:** 10.1080/0886022X.2026.2630507

**Published:** 2026-03-10

**Authors:** Weili Wang, Yilin Gao, Yizhen Chen, Meng Cheng, Liuting Wei, Yonghao Sang, Lei Zhang, Rong Dai, Yiping Wang

**Affiliations:** Department of Nephrology, The First Affiliated Hospital of Anhui University of Chinese Medicine, Hefei, China

**Keywords:** Lactate, renal fibrosis, PPARα, fatty acid oxidation

## Abstract

Chronic kidney disease (CKD) is characterized by renal fibrosis as its core pathological feature, and lipid metabolism disorders are a key driver of disease progression. However, the specific pathological significance of elevated lactate levels in patients with CKD remains unclear. This study aimed to verify the hypothesis that lactate exacerbates renal fibrosis by inhibiting the PPARα/FAO pathway. A total of 15 healthy controls and 75 CKD patients were enrolled. Serum lactate levels were measured, and their correlations with Scr, BUN, eGFR, and lipid metabolism parameters (triglycerides [TG], total cholesterol [TCH]) were analyzed. Meanwhile, unilateral ureteral obstruction (UUO) mouse models and transforming growth factor-β1 (TGF-β1)-induced human proximal tubular epithelial cells (HK-2 cells) were used to validate the regulatory role of lactate in renal fibrosis. Results showed that serum lactate levels in CKD patients significantly increased with disease stage progression, and were positively correlated with Scr, BUN, TG, and TCH (*p* < 0.05), while negatively correlated with eGFR (*p* < 0.0001). RNA sequencing and Western blot confirmed that UUO mouse kidney tissues exhibited lactate accumulation, downregulation of the PPARα/FAO pathway, lipid accumulation, and aggravated renal fibrosis. Exogenous lactate supplementation exacerbated TGF-β1-induced fibrosis and lipid disorders in HK-2 cells, whereas inhibition of lactate production by oxamic acid sodium significantly reversed these pathological effects. In conclusion, lactate disrupts renal lipid homeostasis and exacerbates renal fibrosis by inhibiting the PPARα/FAO pathway. This study provides an important theoretical basis for elucidating the pathological mechanism of CKD and developing novel therapeutic targets.

## 1. Introduction

Chronic kidney disease (CKD) has become a major global public health concern. In 2021, the global prevalence of CKD reached 673.7 million, accounting for 8.54% of the global population—an increase of 92.0% compared to 1990 [1]. There are 434.3 million adult patients in Asia, with those from China and India accounting for 69.1% of the region’s cases, representing the population with the highest disease burden [2]. A progressive decline in renal function is the hallmark of CKD progression. Renal fibrosis, characterized by renal interstitial collagen deposition, renal tubular atrophy, and inflammatory cell infiltration [3–5], is the key pathological change throughout all stages of CKD [4] and the primary driver of the deterioration of renal function to end-stage renal disease [6,7].

Recently, the interaction between metabolic disorders and renal injury has become a focal point in CKD research. Specifically, lipid metabolism imbalance has been confirmed as a key contributor to renal fibrosis [8,9]. Under physiological conditions, the kidneys efficiently utilize fatty acids for energy through fatty acid oxidation (FAO). This process provides the primary energy source for renal tubular epithelial cells and prevents excessive lipid accumulation in the kidneys [10,11]. However, in the context of CKD, renal FAO function is significantly impaired, weakening the fatty acid catabolic capacity of renal tubular epithelial cells and leading to massive intracellular lipid droplet accumulation [12]. The excessive lipids can directly induce oxidative stress, cellular senescence, and apoptosis in renal tubular epithelial cells [13–15], while also aggravate renal fibrosis by activating inflammatory signaling pathways and promoting epithelial-mesenchymal transition (EMT) [16,17].

Peroxisome proliferator-activated receptors (PPARs), members of the ligand-activated nuclear receptor superfamily, are key regulators of FAO [18,19]. Among them, PPARα is the central transcription factor governing the transcription of genes involved in peroxisomal and mitochondrial FAO pathways, fatty acid uptake, and triglyceride (TG) catabolism, thereby maintaining intracellular lipid homeostasis [20–22]. Previous studies have demonstrated that the expression of PPARα is significantly downregulated in patients with CKD and animal models [23,24]. Furthermore, PPARα agonists can effectively delay the progression of renal fibrosis by restoring FAO function, enhancing renal fat catabolism, and reducing lipid accumulation [25,26].

Lactate, a pivotal intermediate product of glucose metabolism, has attracted increasing attention due to its metabolic dysregulation in CKD. Clinical studies indicate significantly elevated blood and urine lactate levels in patients with CKD, correlating with renal function impairment and poor prognosis [27–29]. This phenomenon may stem from dual pathological mechanisms: impaired renal excretion and enhanced anaerobic glycolysis in renal tubular epithelial cells [30,31]. Despite these observations, lactate is still considered as a passive metabolic byproduct. There is no clear evidence regarding whether it actively participates in lipid metabolism imbalance and renal fibrosis by regulating key signaling pathways such as PPARα/FAO.

We hypothesize that lactate disrupts the dynamic balance of renal lipid synthesis and degradation by inhibiting the PPARα signaling pathway and FAO, ultimately aggravating renal fibrosis. To verify this hypothesis, this study first analyzed the correlation between serum lactate levels and renal function as well as lipid metabolism indicators in Patients with CKD. Then, a unilateral ureteral obstruction (UUO) mouse model was used to verify the causal relationship between elevated renal lactate levels, the PPARα/FAO pathway, lipid accumulation, and renal fibrosis. Finally, human proximal tubular epithelial cells (HK-2 cells) were used as an *in vitro* model to elucidate the specific molecular mechanism by which lactate regulates the PPARα/FAO pathway to mediate lipid accumulation and cellular fibrosis. This study offers novel insights into the pathological mechanism of CKD and identifies potential therapeutic targets.

## 2. Material and methods

### 2.1. Chemicals and reagents

Lactate (HY-B2227, purity = 85.12%) and oxamic acid sodium (HY-W013032A, purity = 99.72%) were purchased from MedChemExpress (MO, USA). 1.25% tribromoethanol (2506B) was purchased from Tigergene (Nanjing, China). Human kidney-2 proximal tubule epithelial cells (HK-2 cells, iCell-h096) were obtained from iCell (Shanghai, China). Transforming growth factor-β1 (TGF-β1, CA59) was purchased from Novoprotein (Suzhou, China). Masson’s trichrome staining solution, hematoxylin staining solution, and alcohol-soluble eosin staining solution were acquired from Ebiogo (Hefei, China). Dulbecco’s modified Eagle’s medium (DMEM), fetal bovine serum (FBS), trypsin, phosphate-buffered saline (PBS), and DEPC-H_2_O were purchased from Biosharp (Hefei, China). Antibodies against β-actin (TA-09), goat anti-mouse IgG (ZB-2305), and goat anti-rabbit IgG (ZB-2301) were obtained from Zs-BIO (Beijing, China). Primary antibodies against α-smooth muscle actin (α-SMA, AF1032), fibronectin (FN, AF5335), connective tissue growth factor (CTGF, 15f5788), E-cadherin (E-cad, BF0219), peroxisome proliferator-activated receptor α (PPARα, AF5301), peroxisome proliferator-activated receptor gamma coactivator 1 alpha (PGC1α, AF5395), and sterol regulatory element-binding protein 1 (SREBP1, AF6283) were purchased from Affinity Biosciences (Wuhan, China). The primary antibody against carbohydrate response element-binding protein (CHREBP, BS7083) was obtained from Bioworld (Nanjing, China). The primary antibody against carnitine palmitoyltransferase 1 alpha (CPT1α, HA722081) was purchased from HUABIO (Hangzhou, China). 4′,6-diamidino-2-phenylindole (DAPI) was acquired from Beyotime (Shanghai, China). Bovine serum albumin (BSA) was purchased from Solarbio (Beijing, China). TRIzol reagent was obtained from Share-bio (Shanghai, China). Primer synthesis was performed by Sangon Biotech (Shanghai, China). Taq SYBR Green qPCR Premix (Universal, EG20117M) and All-in-One First-Strand Synthesis MasterMix (with dsDNase, EG15133S) were purchased from iScience (Menlo Park, CA, USA). The Oil Red O staining solution (G1015) and CCK-8 assay kit (GK10001) was obtained from Servicebio (Wuhan, China). The E-Click EdU Cell Proliferation Imaging Assay Kit (Green, Elab Fluor^®^ 488, E-CK-A376) and L-lactic acid (LA) Colorimetric Assay Kit (E-BC-K044-M) were purchased from Elabscience (Wuhan, China). The serum creatinine (Scr) assay kit (C011-2-1), blood urea nitrogen (BUN) assay kit (C013-2-1), total cholesterol (TCH) assay kit (A111-1-1), and triglyceride (TG) assay kit (A110-1-1) were obtained from Njjcbio (Nanjing, China). The Seahorse XF Cell Mito Stress Test Kit (103016-100) was purchased from Agilent Technologies (Santa Clara, CA, USA).

### 2.2. Clinical sample collection

A total of 75 patients diagnosed with CKD who attended the Department of Nephropathy, the First Affiliated Hospital of Anhui University of Chinese Medicine, from December 2024 to June 2025 were recruited as the study group. According to the CKD staging criteria, these patients were stratified into stages 1–5, with 15 cases assigned to each stage. None of the enrolled patients had received renal replacement therapy. The disease spectrum of the CKD group included IgA nephropathy, membranous nephropathy, lupus nephritis, hypertensive nephropathy, diabetic nephropathy, and other subtypes. Additionally, 15 healthy volunteers were concurrently recruited as the normal control group (CTL group). Peripheral blood samples were collected from all subjects in both groups, and subsequent analyses were conducted to compare the differences in serum lactic acid concentrations among CKD patients at different stages. This study was approved by the Research Ethics Committee of the First Affiliated Hospital of Anhui University of Chinese Medicine (approval number: 2022-AH27), permitting the inclusion of human subjects. All subjects signed written informed consent forms in accordance with the study protocol.

### 2.3. Animal model and treatment

SPF grade male C57BL/6 wild-type mice, 8 weeks old and weighing 20–25 g, were purchased from Jiangsu Jicui Yaokang Biotechnology Co., Ltd. (China). All mice were housed in the Experimental Animal Center of Anhui University of Chinese Medicine under constant temperature and humidity conditions, with free access to food and water. After one week of acclimatization, the experiment was initiated. Independent researchers not involved in subsequent experimental operations randomly divided the mice into a sham operation group (Sham group) and a unilateral ureteral obstruction group (UUO group) using a random number table method, with 6 mice in each group, totaling 12 mice. The cages of the two groups were randomly placed on different shelf levels in the animal room, and experimental operations were performed in the order of randomly generated animal numbers.

The UUO model was established as previously described [32]. Briefly, mice were anesthetized intraperitoneally with 1.25% tribromoethanol solution at a dose of 20 mL/kg. A 1 cm longitudinal incision was made on the left abdomen to expose the left kidney and ureter. The ureter was ligated and transected using 4-0 absorbable sutures, followed by layer-by-layer closure of the abdominal incision. The Sham group underwent the same abdominal incision and organ exposure procedures without ureteral ligation and transection, while all other operations were consistent with those of the UUO group. The experimental period was 14 days, during which both groups of mice were maintained under identical housing conditions. At the end of the experiment, mice were euthanized by isoflurane inhalation overdose, and blood and left kidney tissue samples were collected and properly preserved. Each kidney tissue sample was divided into two parts: one for biochemical evaluation and the other for pathological examination. This study protocol was approved by the Ethics Committee of Anhui University of Chinese Medicine (Approval No.: AZYFY-2025-3003). All animal experimental procedures were strictly performed in accordance with the ARRIVE guidelines and the relevant regulations of the Institutional Animal Care and Use Committee (IACUC).

### 2.4. Cell culture and treatment

HK-2 cells were cultured in DMEM containing 10% fetal bovine serum (FBS) and incubated in a humidified incubator at 37 °C with 5% CO_2_. For experiments, HK-2 cells were seeded into 6-well plates or glass slides. The cells were treated with 10 ng/mL TGF-β1 for 24 h, followed by medium change and an additional 24h incubation to induce fibrosis [33]. For intervention experiments, cells were pretreated with lactate (10 mM) or oxamic acid sodium (20 mM) for 24 h [34], followed by treatment with TGF-β1 for 24 h.

### 2.5. Hematoxylin and eosin (H&E) staining and Masson staining

Kidney tissues were fixed in paraformaldehyde, embedded in paraffin, and sectioned at 3 μm. Sections were then subjected to H&E staining and Masson’s trichrome Staining to assess renal injury and fibrosis respectively. Images were captured using a light microscope (OLYMPUS CX43, Center Valley, PA, USA). Three random 400× fields were captured from each section. The positive staining area was quantitatively analyzed using Image J software (NIH, Bethesda, MD, USA).

### 2.6. Immunohistochemistry

Paraffin-embedded sections of kidney tissues were processed according to the manufacturer’s protocols, followed by antigen retrieval and peroxidase blocking. The sections were then incubated with specific primary antibodies against α-SMA and E-cad at 4 °C overnight. After washing steps, the sections were incubated with secondary antibodies, followed by DAB staining and counterstaining with hematoxylin. Quantitative analysis was performed using the method described above.

### 2.7. Oil Red O staining

After sample treatment, the cells or tissues were fixed in 4% formaldehyde for 10 min. Oil Red O staining solution was used for the specific staining of lipid droplets, while DAPI staining solution was applied for nuclear counterstaining. After staining, a fluorescence microscope was used to observe the accumulation of lipid droplets in the cells or tissues, and the positive areas of lipid droplets were quantitatively analyzed using ImageJ image analysis software.

### 2.8. Serum biochemical analysis

The levels of Scr, BUN, TCH, and TG in mouse serum were measured with commercial ELISA kits following the manufacturer’s protocols.

### 2.9. Cell viability assay

Cell viability was assessed using the CCK-8 assay. Briefly, 10 μL of CCK8 reagent was added to a 96-well plate. Cells were cultured for another 1h and the absorbance was measured at a wavelength of 450 nm using a microplate reader. The blank control wells only contain medium and CCK8 reagent. Cell viability was calculated using the formula: Cell viability % = [(As-Ab)/(Ac-Ab)] × 100%, where: As = experimental OD value; Ab = blank OD value; Ac = control OD value.

### 2.10. 5-Ethynyl-2′-deoxyuridine (EdU) proliferation assay

Cell proliferation was evaluated using an EdU imaging kit according to the manufacturer’s instructions. Briefly, cells were incubated with 10 μM EdU dye for 2 h and stained with DAPI. After staining, proliferating cells were labeled with EdU, which emitted bright green fluorescence. The percentage of EdU-positive cells was determined from fluorescence images.

### 2.11. RNA-seq

Total RNA was extracted from renal tissue samples using TRIzol reagent. The integrity of RNA samples was assessed by a Qsep400 automatic nucleic acid and protein analyzer. RNA sequencing was performed by Wuhan Igenebook Biotechnology Co., Ltd. (http://www.igenebook.com) on the Illumina NovaSeq 6000 platform. Raw image data were converted into Raw Reads *via* Base Calling. The raw data were subjected to quality control using FastQC (v0.11.5) and low-quality reads were removed to obtain valid reads. The number of reads was counted using featureCounts (v1.6.0) combined with genome annotation files, and normalized by fragments per kilobase of exon per million reads mapped (FPKM) to enable horizontal comparison of gene expression levels between samples. Differential expression analysis was performed using the R package edgeR (v3.34.0). Significantly differentially expressed genes (DEGs) were identified with the screening thresholds of |log2 fold change (FC)| > 1 and false discovery rate (FDR) < 0.05. Pathway enrichment analysis was performed using kyoto encyclopedia of genes and genomes (KEGG) database.

### 2.12. Western blot

Proteins were extracted from renal tissues or HK-2 cells using RIPA lysis buffer. Protein concentration was determined using a BCA kit. Equal amounts of protein were separated by SDS-PAGE and transferred to nitrocellulose (NC) membranes. Subsequently, the membranes were incubated with primary antibodies at 4 °C overnight, followed by HRP-conjugated secondary antibodies. Finally, protein bands were quantified using Image J software.

### 2.13. Reverse transcription quantitative polymerase chain reaction (RT-qPCR)

Total RNA was extracted using Trizol, and reverse-transcribed into cDNA using the All-in-One First-Strand Synthesis MasterMix kit. RT-qPCR was performed using Taq SYBR Green qPCR Premix. Relative gene expression was calculated using the 2^−ΔΔCt^ method and normalized to β-actin. Primer sequences are listed in Table S1.

### 2.14. Determination of lactate content

Frozen kidney tissues or cells were homogenized in PBS on ice, then centrifuged at 12,000 rpm at 4 °C for 15 min. The lactate in the supernatant was measured using the L-lactic acid colorimetric assay kit according to the manufacturer’s instructions.

### 2.15. Measurement of oxygen consumption rate (OCR)

OCR was measured using a Seahorse XFe96 Extracellular Flux Analyzer (Agilent Technologies, Santa Clara, CA, USA). Briefly, HK-2 cells were seeded into Agilent 96-well plates and incubated overnight, followed by different treatments prior to the assay. OCR analysis was performed according to the instructions of the Seahorse XF Cell Mito Stress Test Kit. Results were analyzed using Seahorse XF 96 Wave software and expressed as pmol/min.

### 2.16. Statistical analysis

All statistical analyses, including spearman correlation analysis, one-way analysis of variance (ANOVA), multiple comparisons, and Student’s t-test, were performed using GraphPad Prism 10.0 software. Data are expressed as mean ± standard error of the mean (SEM). A *p*-value < 0.05 was considered statistically significant.

## 3. Results

### 3.1. Serum lactate levels are associated with lipid metabolism disorders in patients with CKD

**Figure 1.**
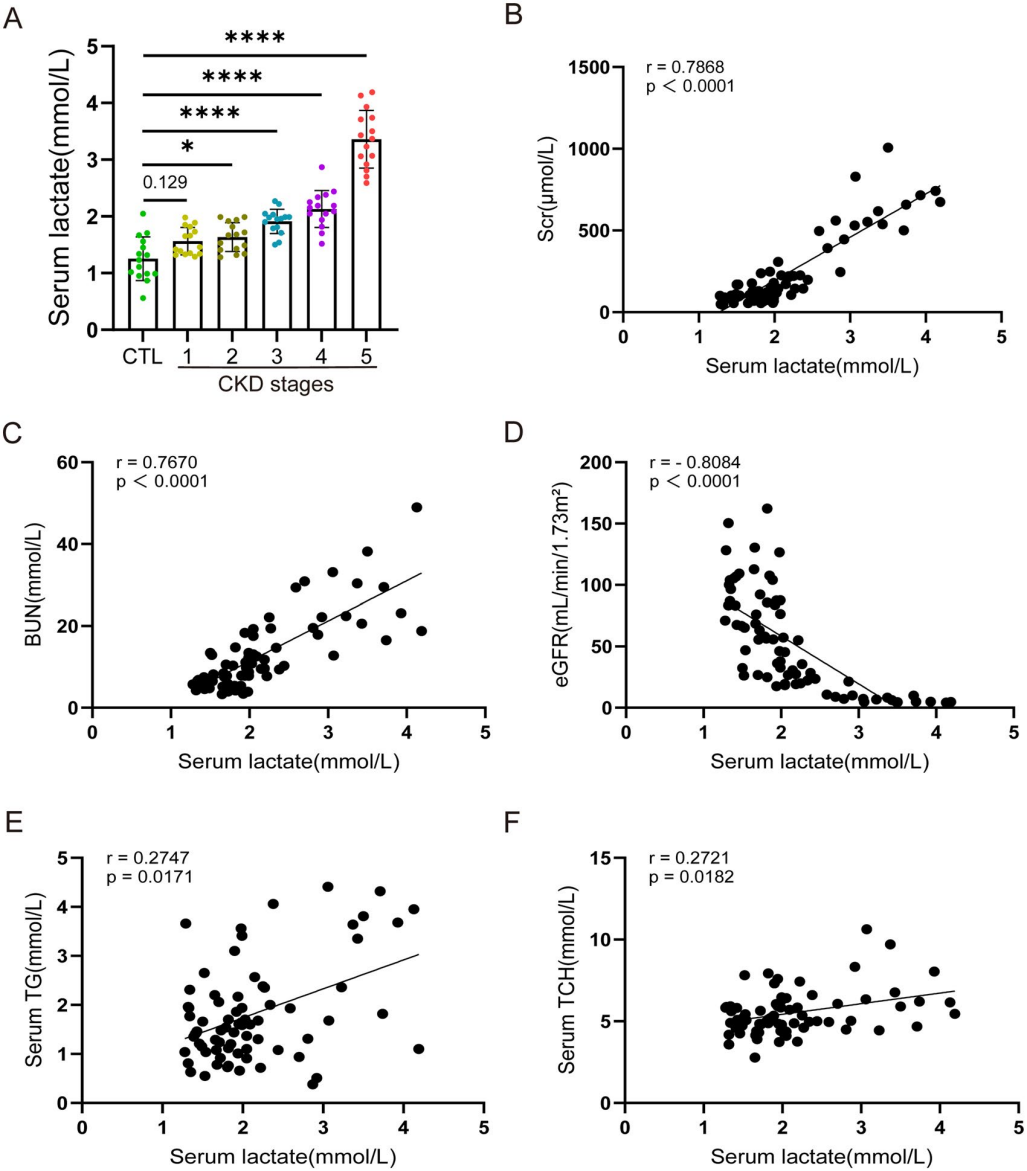
Lactate levels are associated with lipid metabolism in patients with CKD. (A) Quantitative analysis of serum lactate levels in different stages of CKD (*n* = 75). *p < 0.05 vs CTL, **p < 0.01 vs CTL, ***p < 0.001 vs CTL, ****p < 0.0001 vs CTL. (B − F) Correlations between serum lactate levels and Scr (B), BUN (C), eGFR (D), serum TG (E), and serum TCH (F) in patients with CKD (*n* = 75). Data are expressed as mean ± standard error of the mean (SEM). Correlation analysis was performed using spearman correlation analysis.

To explore the association between lactate levels and CKD, this study first analyzed the serum lactate levels across different CKD stages. The serum lactate levels were significantly higher in patients with CKD than those in the control group, and exhibited a stepwise increase from CKD stages 1–5 (Figure 1A). Serum lactate levels were positively correlated with Scr and BUN levels, but negatively correlated with eGFR (*p* < 0.0001, Figure 1B–D). Furthermore, we evaluated the correlation between serum lactate levels and lipid metabolism parameters in patients with CKD, including serum TG and serum TCH. Our results showed that serum lactate levels were positively correlated with serum TG and TCH (*p* < 0.0001 and *p* = 0.0021, respectively; Figure 1E,F). These results suggest a potential link between serum lactate levels and lipid metabolism disorders in CKD.

### 3.2. Increased renal lactate production accompanies lipid accumulation in UUO mice

**Figure 2.**
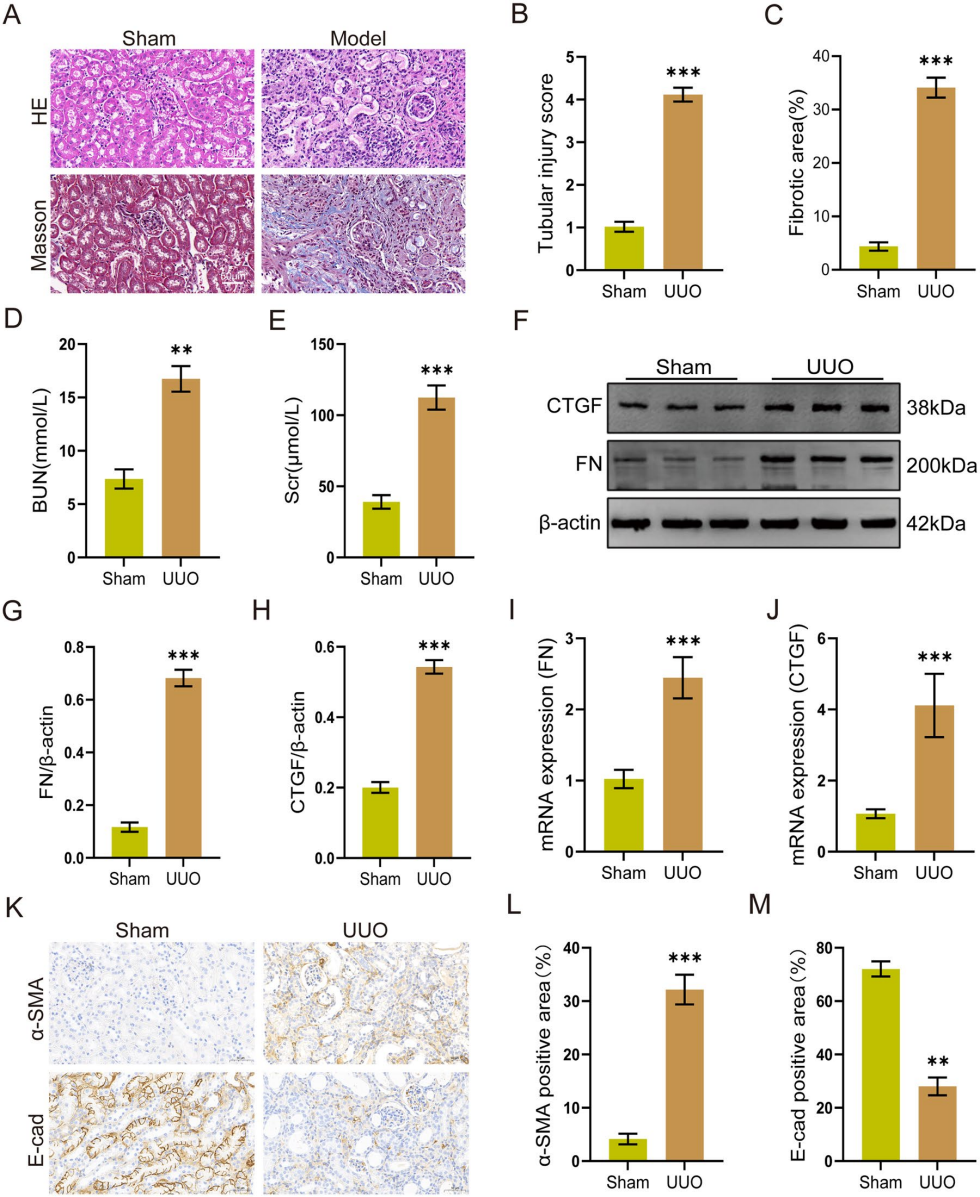
Impaired renal function and renal tissue fibrosis in UUO mice. (A − C) H&E staining and Masson staining in each group (*n* = 6). Scale bar = 50 μm. (D and E) BUN and Scr levels in each group (*n* = 6). (F − H) Protein expression levels of FN and CTGF in renal tissues (*n* = 3). (I and J) mRNA expression levels of FN and CTGF in renal tissues (*n* = 6). (K − M) Immunohistochemical staining and quantification for the expression levels of α-SMA and E-cad in renal tissues (*n* = 3). Scale bar = 50 μm. **p < 0.01 vs Sham, ***p < 0.001 vs Sham.

We utilized the UUO model to investigate the effects of lactate on renal pathology. Histological analysis *via* H&E and Masson staining showed significant renal tubular injury and fibrosis compared with the Sham group (*p* < 0.001, Figure 2A–C). The levels of Scr (*p* < 0.001) and BUN (*p* < 0.01) were markedly higher in the UUO group, indicating impaired renal function (Figure 2D,E). Results of Western blot and RT-qPCR showed that the expressions of CTGF and FN in renal tissues of the UUO group were increased (*p* < 0.001, Figure 2F–J). Immunohistochemical results further indicated that the expression of E-cad (*p* < 0.01) was downregulated and the expression of α-SMA (*p* < 0.001) was upregulated in renal tissues of the UUO group (Figure 2K–M). These results further confirmed the elevated renal fibrosis in UUO mice.

**Figure 3.**
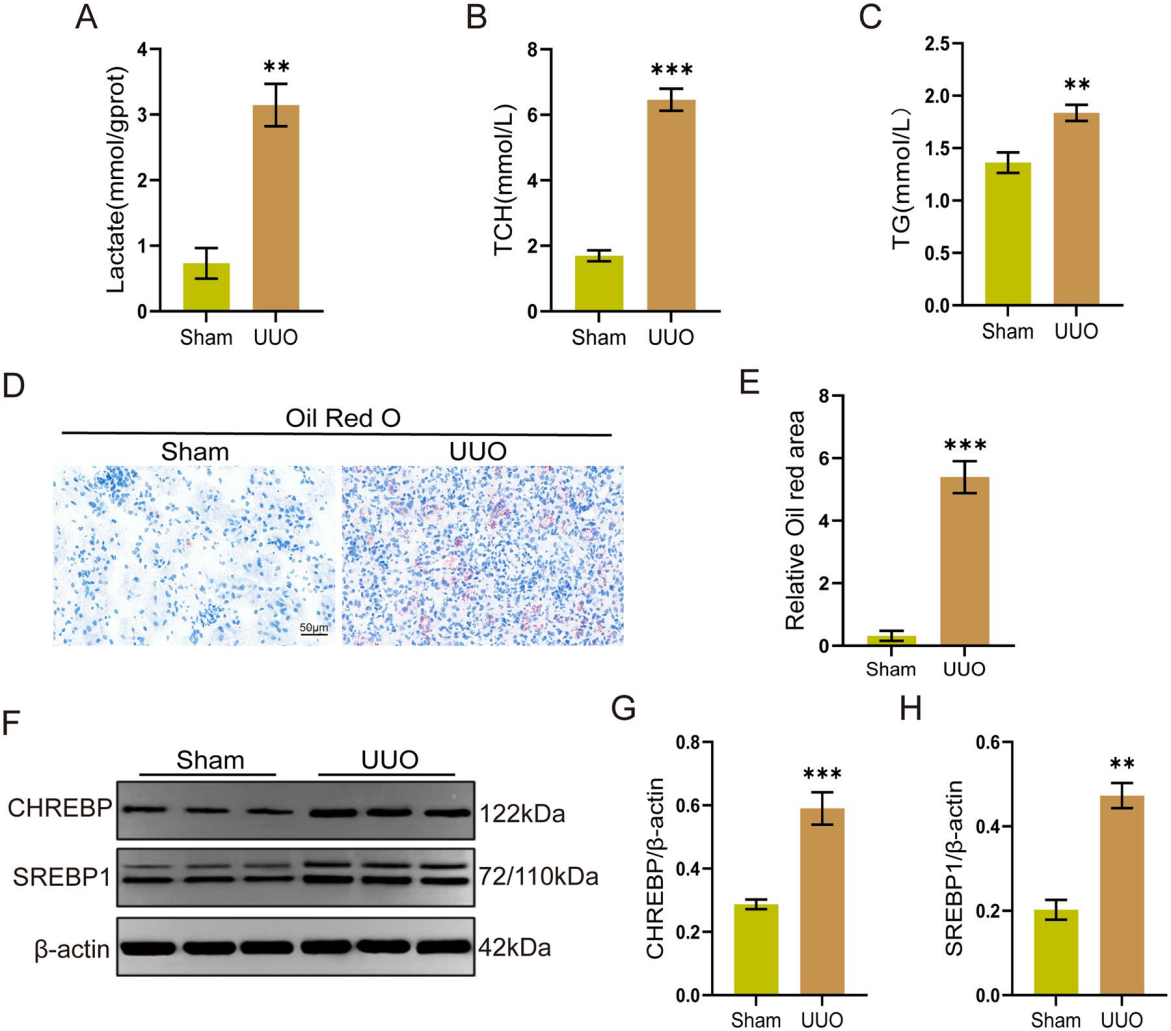
Elevated lactate levels and lipid metabolism disorders in UUO mice. (A) Renal tissue lactate levels (*n* = 6). (B and C) Serum TG and TCH levels (*n* = 6). (D and E) Representative images and quantitative analysis of Oil Red O staining (*n* = 3). Scale bar = 50 μm. (F − H) Protein expression levels of CHREBP and SREBP1 (*n* = 3). ** *p* < 0.01 vs Sham, ***p < 0.001 vs Sham.

We further analyzed the lactate levels in renal tissues of UUO mice. Notably, lactate levels in renal tissues were significantly increased in of UUO mice (*p* < 0.01, Figure 3A), accompanied by elevated renal serum TG (*p* < 0.01) and serum TCH (*p* < 0.01) content (Figure 3B,C). Oil Red O staining showed substantisl renal lipid accumulation in fibrotic kidneys (*p* < 0.001, Figure 3D,E). CHREBP, a glucose-responsive transcription factor, promotes the conversion of glucose to lipids and the accumulation of intracellular triglycerides. SREBP1 acts as a master transcription factor in lipid synthesis; its activation drives the biosynthesis of fatty acids and triglycerides, and these two factors cooperatively regulate the key pathways of cellular lipid synthesis [35]. To further verify the expression profiles of these two factors in renal lipid metabolism, we detected the expression levels of related proteins in renal tissues of UUO model mice *via* Western blot assays. The results showed that the expression levels of both CHREBP (*p* < 0.001) and SREBP1 (*p* < 0.001) in renal tissues were significantly upregulated (Figure 3F–H). These results indicate that elevated lactate levels are accompanied by dyslipidemia and renal lipid accumulation during renal fibrosis in UUO mice.

### 3.3. RNA-seq reveals abnormal lipid metabolism-related pathways in UUO mice

**Figure 4.**
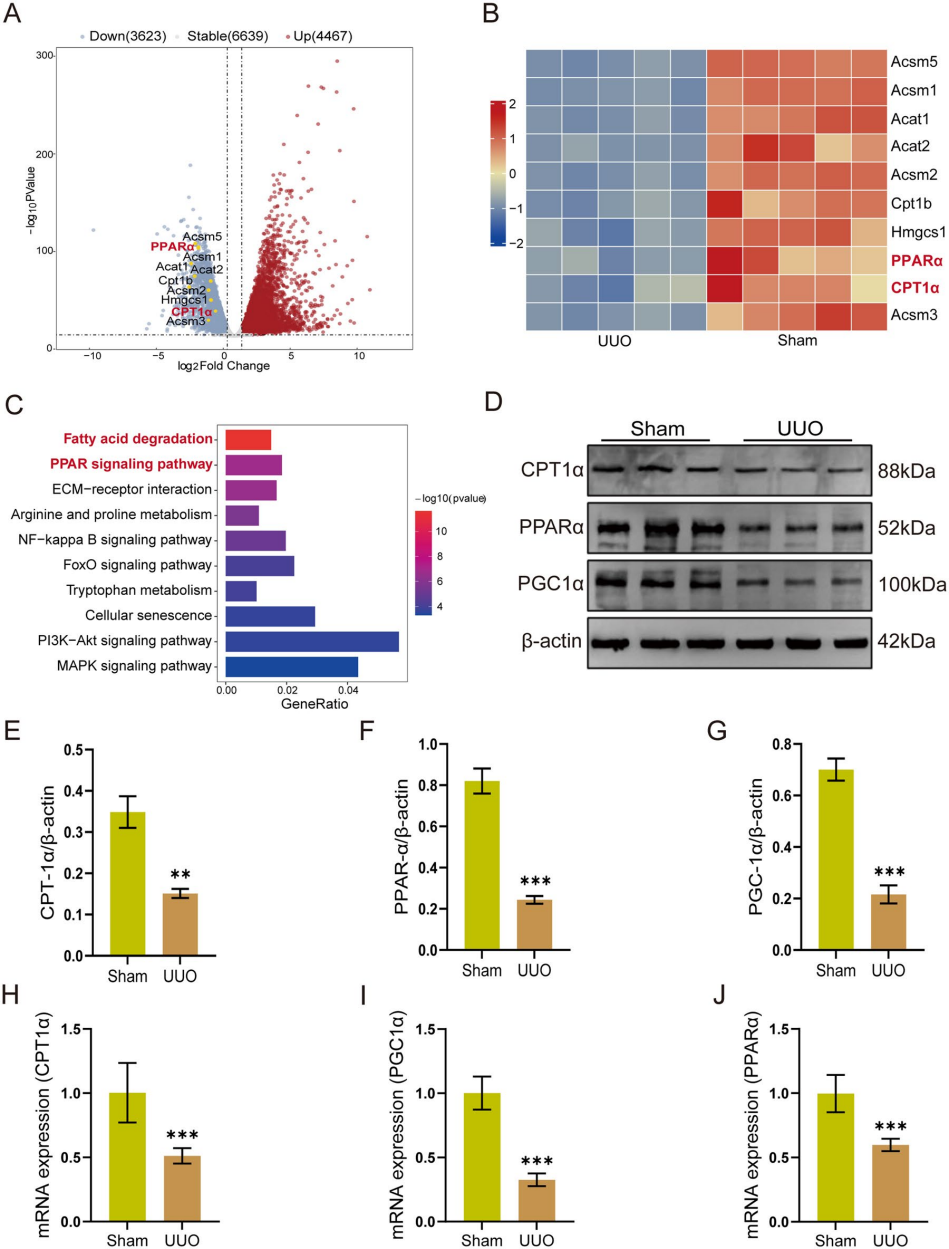
Suppression of the PPAR pathway and fatty acid metabolism in renal tissues of UUO mice. (A) Volcano plot of DEGs from RNA-seq. (B) Heatmap of differentially expressed genes between the UUO group and the Sham group. (C) KEGG enrichment analysis of differentially downregulated genes. (D − J) Protein and mRNA expression levels of *PPARα*, *PGC1α*, and *CPT1α* in each group (*n* = 6). **p < 0.01 vs Sham, ***p < 0.001 vs Sham.

Our analysis of RNA-seq data identified distinct gene expression patterns between Sham and UUO kidneys. We observed significant changes in several lipid metabolism-related genes, including PPARα, CPT1α, CPT1b, Acsm5, Acsm1, Acsm2, Hmgcs2, Acat1, and Acat2 (Figure 4A). Particularly, the expressions of PPARα, CPT1α, and Acat2 in UUO mice were significantly decreased (Figure 4B). KEGG analysis revealed that these differentially downregulated genes were significantly enriched in the fatty acid degradation and PPAR signaling pathway (Figure 4C). Given that the PPARα pathway serves as the core regulatory pathway for FAO and global lipid metabolism, and PPARα acts as a key transcription factor that directly governs the expression and regulation of FAO-related genes, we subsequently focused on validating the PPARα pathway and its downstream associated molecules. Results from Western blot and RT-qPCR assays demonstrated that the protein and mRNA expression levels of CPT1α, PPARα and PGC1α in the UUO group were significantly downregulated (*p* < 0.01, *p* < 0.001, Figure 4D–J). It should be noted that FAO represents the core metabolic process for fatty acid degradation, while PPARα is the central transcription factor regulating FAO; its downregulation directly inhibits the expression of CPT1α, a critical enzyme in the FAO cascade. Moreover, PGC1α functions as a coactivator of PPARα, and its reduced expression further impairs the transcriptional activity of PPARα, ultimately leading to the attenuation of FAO function [36]. Taken together, these findings suggest that UUO treatment may induce lipid metabolic disorder in mice by interfering with the PPARα-FAO axis, and such metabolic abnormalities may contribute to the progression of UUO-induced renal fibrosis.

### 3.4. Exogenous lactate exacerbates TGF-β1-induced fibrosis in HK-2 cells

**Figure 5.**
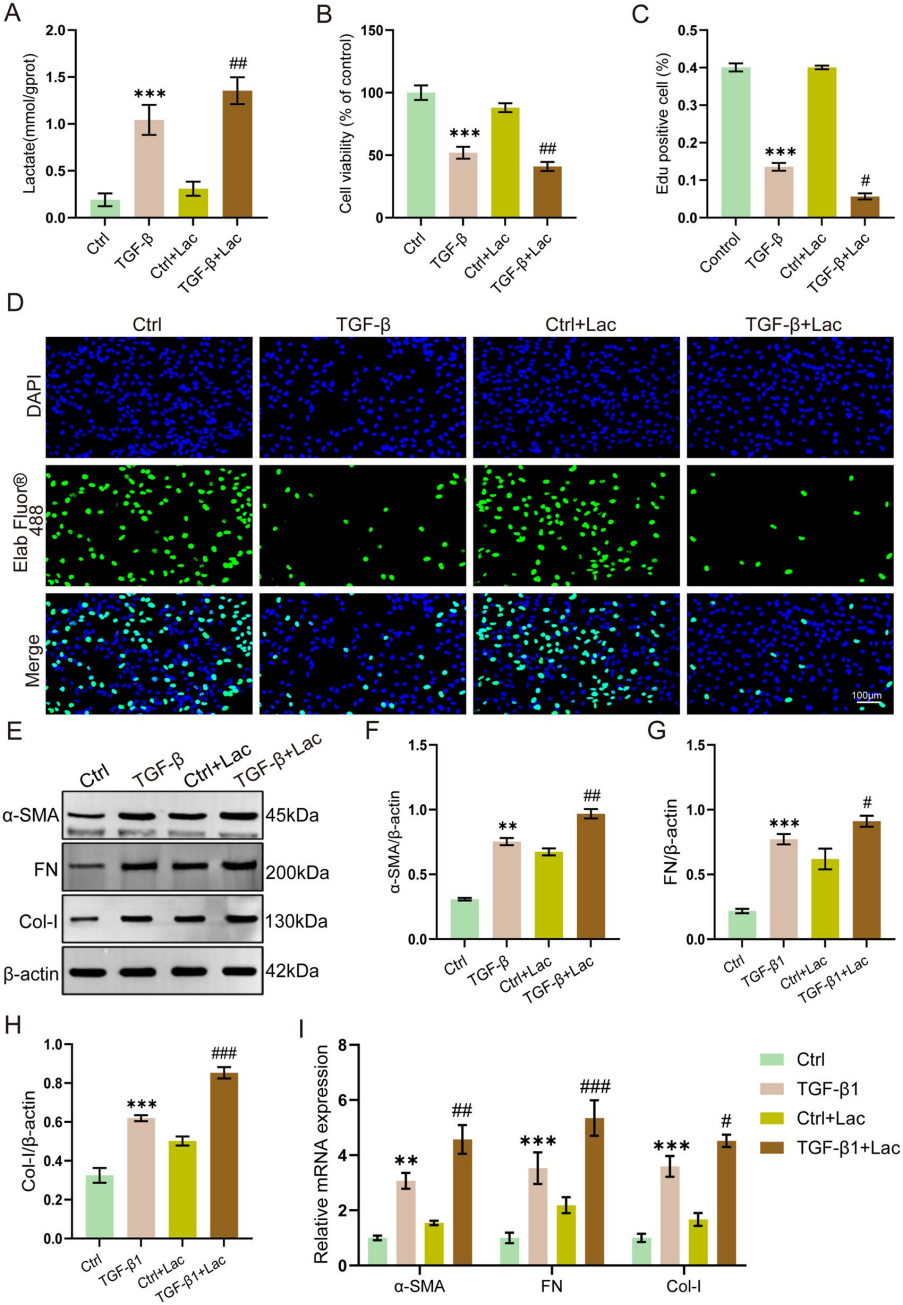
Exogenous lactate exacerbates TGF-β1-induced fibrosis in HK-2 cells. (A) Lactate levels in HK-2 cells (*n* = 6). (B) Cell viability (*n* = 6). (C and D) EdU cell proliferation assay quantification and representative images (*n* = 3). Scale bar = 100 μm. (E − H) Western blot analysis of α-SMA, FN and Col-I (*n* = 3). (I) mRNA level of α-SMA, FN and Col-I (*n* = 6). **p < 0.01 vs Control, ***p < 0.001 vs Control, #*p* < 0.05 vs TGF-β, ##*p* < 0.01 vs TGF-β, ###*p* < 0.001 vs TGF-β.

To investigate whether lactate promotes fibrosis, we treated TGF-β1-stimulated HK-2 cells with 10 mM lactate. In the results, exogenous lactate significantly increased intracellular lactate levels (*p* < 0.01, Figure 5A) and further reduced cell viability compared with the TGF-β1 treatment along (*p* < 0.01, Figure 5B). EdU assay showed that TGF-β1 treatment significantly suppressed cell proliferation, while lactate treatment further exacerbated TGF-β1-induced suppression of cell proliferation (Figure 5C,D).

To clarify the effect of exogenous lactate on TGF-β1-induced fibrosis in HK-2 cells, we analyzed the expression levels of the fibrotic marker α-SMA, FN, and Collagen Type I (Col-I). Western blot and RT-qPCR data demonstrated that lactate co-treatment further enhanced the TGF-β1-induced expression of α-SMA, FN, and Col-I at both the protein and mRNA levels (Figure 5E–I).

### 3.5. Lactate inhibits the PPARα/FAO signaling pathway and promotes lipid accumulation in HK-2 cells

**Figure 6.**
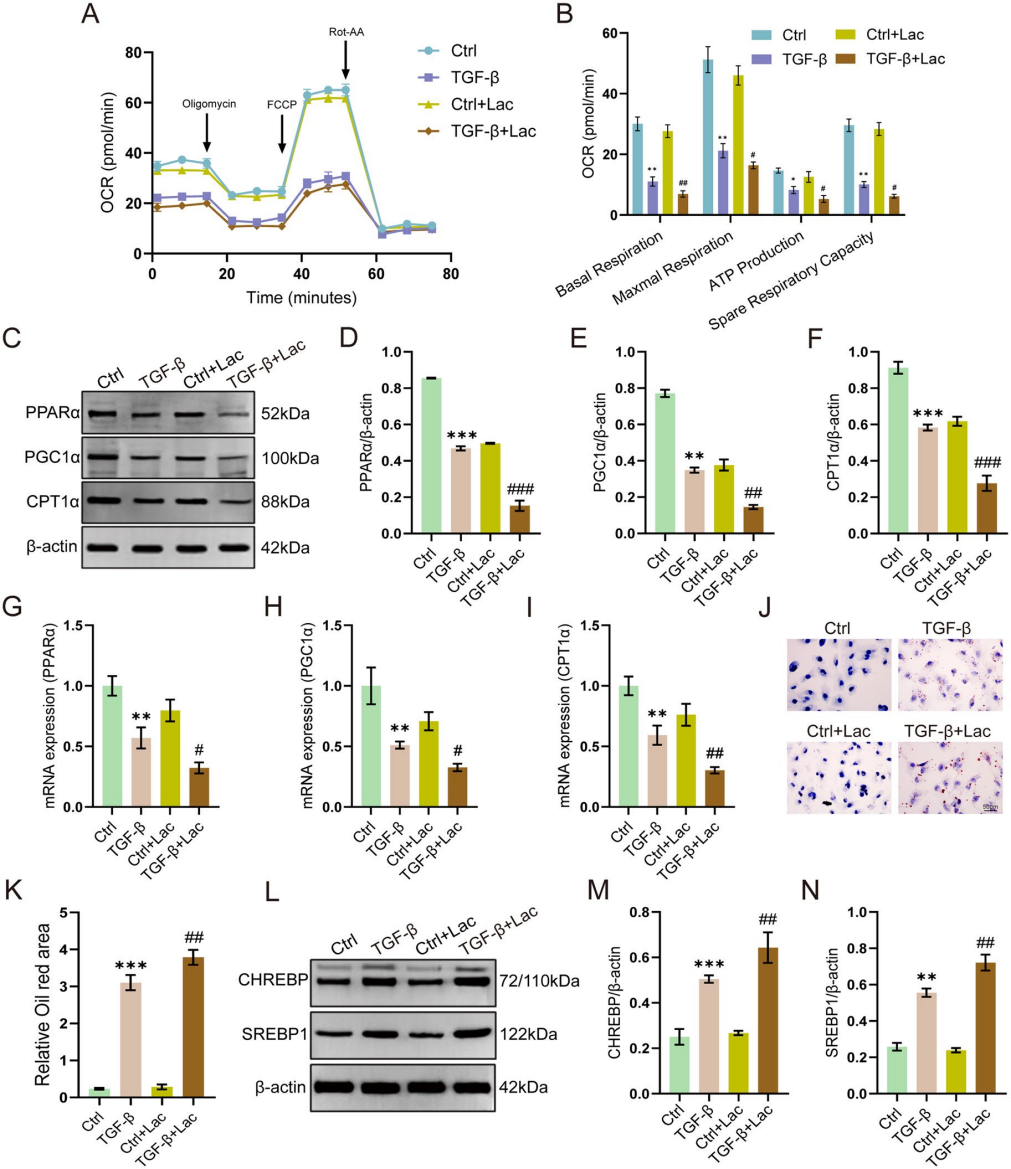
Lactate inhibits the PPAR/FAO pathway and promotes lipid accumulation in HK-2 cells. (A and B) Mitochondrial stress test profiles and OCR quantification (*n* = 3). (C − F) Western blot analysis of PPARα, PGC1α, and CPT1α (*n* = 3). (G − I) mRNA expression of *PPARα*, *PGC1α*, and *CPT1α* (*n* = 6). (J and K) Representative Oil Red O staining and quantitative analysis. Scale bar = 50 μm. (L − N) Western blot analysis of CHREBP and SREBP1 (*n* = 3). *p < 0.05 vs Control, **p < 0.01 vs Control, ***p < 0.001 vs Control, #*p* < 0.05 vs TGF-β, ##*p* < 0.01 vs TGF-β, ###*p* < 0.001 vs TGF-β.

We next investigate the metabolic effect of lactate on HK-2 cells using Seahorse analysis. The results showed that TGF-β1 treatment suppressed the OCR levels in HK-2 cells, while exogenous lactate supplementation further exacerbated the effect (Figure 6A,B). Given the important role of FAO in mitochondria respiration, we further determined levels of key enzymes involved in FAO. The results showed that lactate treatment further downregulated expression of PPARα, PGC1α, and CPT1α in the TGF-β1-treated cells (Figure 6C–I).

Concurrently, lactate aggravated intracellular lipid accumulation, as evidenced by Oil Red O staining (Figure 6J,K) and the increased expression of lipogenic proteins CHREBP and SREBP1 (Figure 6L–N). These results suggest that lactate aggravates lipid accumulation by inhibiting FAO and promoting lipogenesis in TGF-β1-stimulated HK-2 cells.

### 3.6. Inhibition of lactate production alleviates TGF-β1-induced fibrosis in HK-2 cells

**Figure 7.**
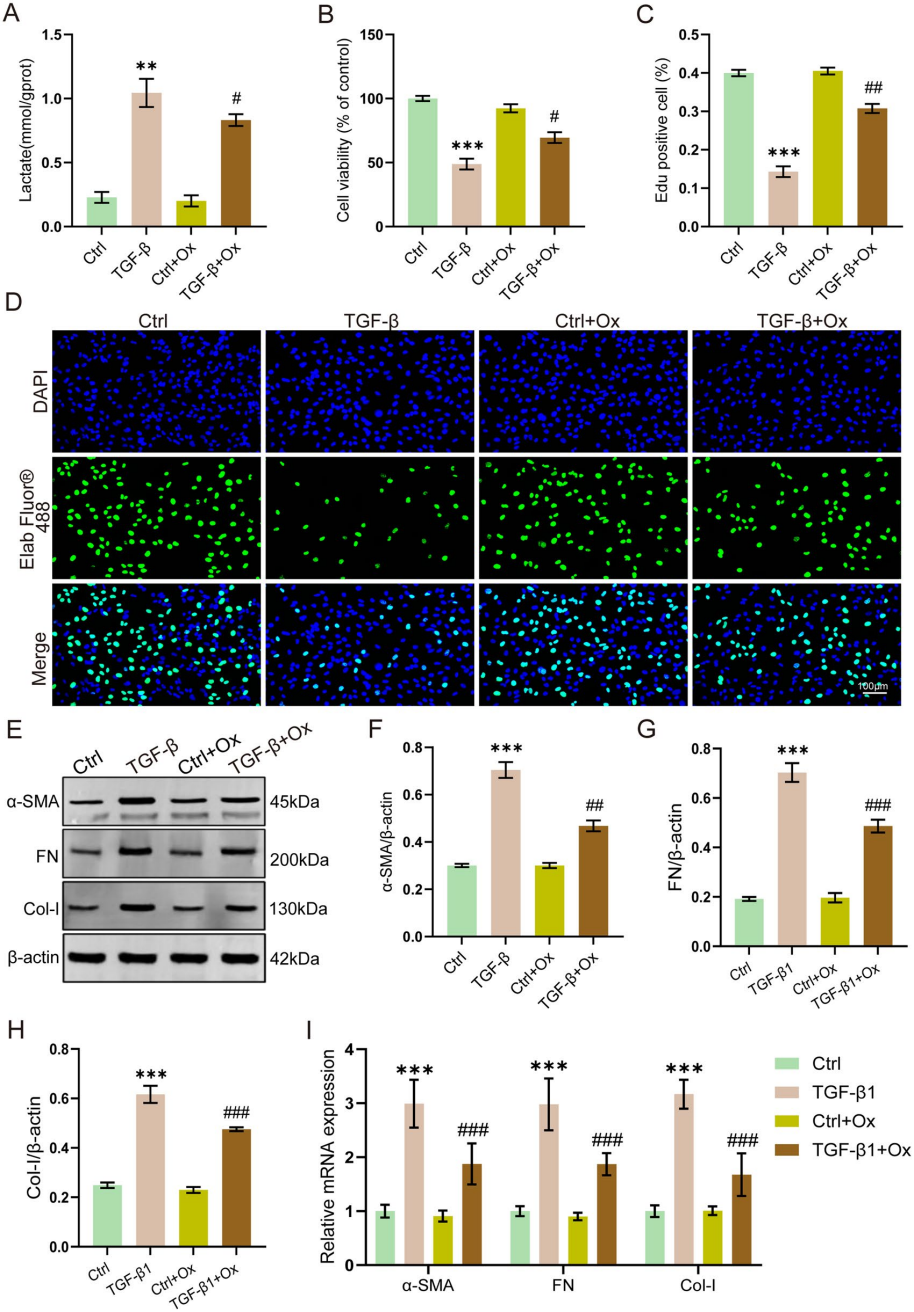
Lactate inhibition alleviates TGF-β1-induced fibrosis in HK-2 cells. (A) Lactate levels in HK-2 cells (*n* = 6). (B) Cell viability (*n* = 6). (C and D) EdU cell proliferation assay quantification and representative images (*n* = 3). Scale bar = 100 μm. (E − H) Western blot analysis of α-SMA, FN and Col-I (*n* = 3). (G) mRNA expression of α-SMA, FN and Col-I (*n* = 6). **p < 0.01 vs Control, ***p < 0.001 vs Control, #*p* < 0.05 vs TGF-β, ##*p* < 0.01 vs TGF-β.

To investigate whether inhibition of lactate production can alleviate TGF-β1-induced fibrosis, we treated the cells with oxamic acid sodium (Ox, 20 mM), a lactate dehydrogenase A inhibitor. The results showed that Ox treatment significantly reduced intracellular lactate levels (*p* < 0.05, Figure 7A) and rescued the cell viability in TGF-β1-treated HK-2 cells (*p* < 0.05, Figure 7B). EdU assay also showed that Ox treatment also partially rescued the TGF-β1-suppressed cell proliferation (Figure 7C,D). Furthermore, results from Western blot and RT-qPCR assays demonstrated that Ox inhibited the expression of the fibrotic genes α-SMA, FN, and Col-I in TGF-β1-stimulated HK-2 cells (Figure 7E–I). These results suggest that inhibition of lactate production can effectively reduce TGF-β1-induced injury and fibrosis in HK-2 cells.

### 3.7. Inhibition of lactate production restores the PPARα/FAO pathway and reduce lipid accumulation in TGF-β1-stimulated HK-2 cells

**Figure 8.**
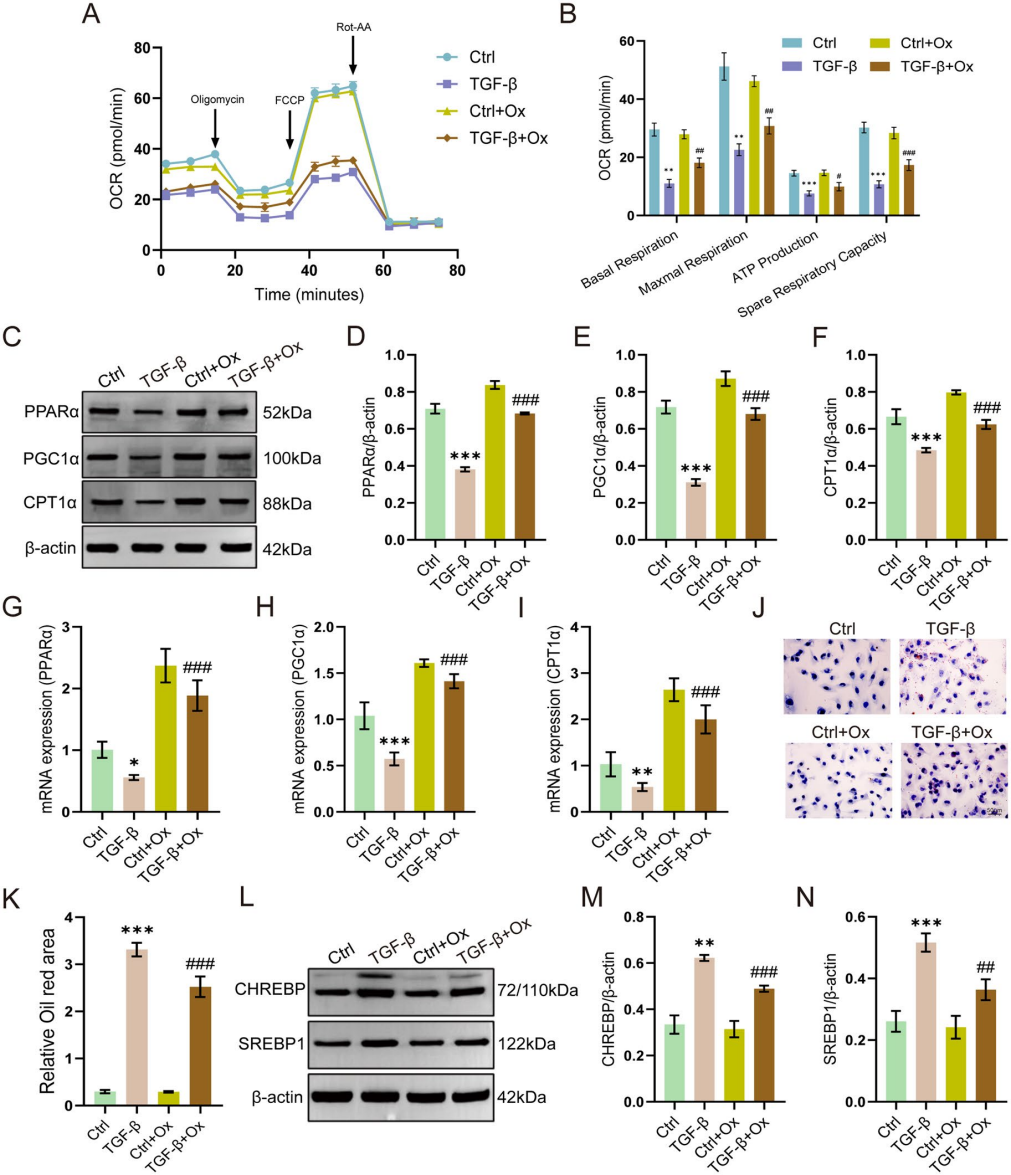
Lactate inhibition restores the PPAR/FAO signaling pathway and reduces lipid accumulation in HK-2 cells. (A and B) OCR profiles and quantification (*n* = 3). (C − F) Western blot analysis of PPARα, PGC1α, and CPT1α (*n* = 3). (G − I) mRNA expression of *PPARα*, *PGC1α*, and *CPT1α* (*n* = 6). (J and K) Representative Oil Red O staining and quantitative analysis (*n* = 3). Scale bar = 50 μm. (L − N) Western blot analysis of CHREBP and SREBP1 (*n* = 3). *p < 0.05 vs Control, **p < 0.01 vs Control, ***p < 0.001 vs Control, ##*p* < 0.01 vs TGF-β, ###*p* < 0.001 vs TGF-β.

Finally, we explored whether reduced lactate levels can restore metabolic homeostasis in HK-2 cells. Ox treatment significantly recovered OCR levels in TGF-β1-stimulated cells (Figure 8A,B). The metabolism restoration was accompanied by upregulated expression of PPARα pathway- and FAO-related genes (PPARα, PGC1α, CPT1α) at both protein and mRNA levels (*p* < 0.001, Figure 8C–I). Moreover, Ox treatment reduced lipid accumulation (Figure 8J,K) and suppressed the expression of lipogenesis-related proteins CHREBP and SREBP1 in TGF-β1-treated HK-2 cells (Figure 8L–N).

These results suggest that lactate inhibition can reactivate the PPARα pathway and FAO, and effectively inhibit TGF-β1-induced lipid accumulation and lipid metabolism disorders in HK-2 cells.

## 4. Discussion

During the progression of CKD, renal fibrosis is the core pathological feature leading to irreversible decline in renal function [37,38]. Metabolic disorders, including dyslipidemia, are considered key drivers accelerating this process [39]. Although the kidney is not recognized as a classical core metabolic organ, the energy supply of renal tubular epithelial cells is highly dependent on the FAO pathway. Cellular senescence can induce abnormal expression of key enzymes such as phospholipase A_2_ and sphingomyelin phosphodiesterase 1 in renal tissues, thereby triggering lipid composition remodeling and FAO functional impairment [40,41]. In turn, the inhibition of FAO function further promotes abnormal lipid accumulation in renal tissues [23]. During the progression of CKD, multiple lipid metabolic pathways including FAO, lipid synthesis, cholesterol metabolism, and sphingolipid metabolism exhibit significant abnormalities. The dysfunction of key regulatory factors such as CPT1α, PPARs, and SREBPs represents a crucial mechanism mediating abnormal lipid deposition and lipotoxic injury in renal tissues [42]. Among these regulators, PPARα, as a core transcription factor governing the FAO pathway, has its functional abnormalities closely associated with lipid metabolic disorders under CKD conditions. However, the upstream factors regulating the PPARα/FAO pathway remain incompletely understood. For the first time, this study systematically reveals that lactate, as a metabolic intermediate, can disrupt the balance between renal lipid synthesis and degradation by inhibiting the PPARα/FAO pathway, ultimately exacerbating renal fibrosis. These findings provide novel insights from a metabolic perspective for the pathological interpretation of CKD.

Our clinical data reveals a robust correlation between serum lactate and CKD severity. This study also exhibits significant correlations between serum lactate with core indicators of renal function and key parameters of lipid metabolism. This aligns previous studies of elevated urinary lactate/creatinine ratio in patients with CKD [43]. Crucially, this study further clarifies that lactate is not merely a simple metabolic end product but forms a pathological interaction with lipid metabolism disorders.

Using the UUO mice model, this study confirmed that renal lactate levels are significantly correlated with the degree of fibrosis, lipid accumulation, and inhibition of the PPARα/FAO pathway. In renal tissues of UUO mice, increased lactate content was accompanied by elevated levels of TG and TCH, as well as lipid accumulation. Western blot and RT-qPCR analysis further confirmed that the expression of lipogenesis-related markers CHREBP and SREBP1 was increased in renal tissues of UUO mice. Furthermore, the expression of fibrotic markers α-SMA, FN and Col-I was significantly upregulated in renal tissues of UUO mice, suggesting that lactate may be involved in exacerbating renal fibrosis. RNA-seq analysis revealed that the expression of PPARα and its downstream key FAO molecules was significantly downregulated in renal tissues of UUO mice. KEGG enrichment analysis further pinpointed that differentially expressed genes were mainly enriched in the PPARα signaling pathway and fatty acid degradation pathway, providing a mechanistic basis for the observed renal lipid disorders in UUO mice.

Mechanistically, *in vitro* experiments further demonstrated that exogenous lactate treatment can exacerbate TGF-β1-induced fibrosis in HK-2 cells, while inhibition of lactate production significantly reverses these effects. Lactate exerts its effects by inhibiting the PPARα signaling pathway and FAO function. It impairs oxidative phosphorylation, downregulates the expression of key FAO enzymes such as PPARα, PGC1α, and CPT1α, and enhances lipogenic factors including CHREBP and SREBP1. Notably, the inhibitory effect of lactate on PPARα may involve transcriptional regulation, post-translational modification or serving as a signaling molecule to regulate gene expression [44]. We hypothesize that lactate may inhibit the transcriptional activity of PPARα target genes by interfering with the nuclear localization of PPARα.

This study is the first to demonstrate that lactate plays an active regulatory role in renal fibrosis. Lactate inhibits FAO, leading to an energy metabolism imbalance and insufficient energy supply to renal tubular epithelial cells. Lactate also promotes lipid synthesis, triggering lipotoxic damage, and ultimately inducing EMT and extracellular matrix deposition. Our findings provide novel insights into the understanding of metabolic disorders in CKD. Lactate bridges the abnormal glucose metabolism (lactate accumulation) and abnormal lipid metabolism (FAO inhibition) through the PPARα pathway, jointly driving fibrosis.

This study still has certain limitations: first, clinical samples only analyzed the correlation between serum lactate and systemic lipid metabolism. Due to the scarcity of renal biopsy tissue samples and ethical constraints, it failed to directly detect the direct association between local renal lactate levels and tissue lipid deposition in patients with CKD. Second, the UUO model is an obstructive nephropathy model, and its pathophysiological characteristics differ from those of other CKD etiologies such as diabetic nephropathy and glomerulonephritis. Thus, the confirmation in diabetic or glomerular disease models is warranted. Third, oxamic acid sodium, the LDHA inhibitor adopted in this study, may have certain off-target effects. Future studies could combine metabolomics techniques to elucidate the dynamic changes of renal lactate metabolic pathways in patients with CKD. Additionally, LDHA gene knockdown and PPARα gene knockdown techniques can be employed to clarify the indispensable role of lactic acid in regulating renal fibrosis.

## 5. Conclusion

This is the first study to demonstrate that lactate exacerbates renal lipid accumulation by inhibiting the PPARα signaling pathway and FAO, ultimately aggravating renal fibrosis. Our findings reveal a novel role of lactate as a metabolic signaling molecule connecting glucose and lipid metabolism and driving renal fibrosis. It provides an important theoretical basis for the pathological interpretation and targeted therapy of CKD.

## Supplementary Material

Supplemental Material

## Data Availability

All data generated and described in this article are available from the corresponding author on reasonable request.
