# Supplementary material for: Lactate induces renal lipid accumulation and aggravates renal fibrosis by inhibiting the PPARα signaling pathway and fatty acid oxidation
Source: Ren Fail. 2026 Mar 10;48(1):2630507. doi: 10.1080/0886022X.2026.2630507 (PMC12981269; doi:10.1080/0886022X.2026.2630507)
Supplement: Supplemental Material [file IRNF_A_2630507_SM6682.docx]

**Table S1.** The list of primer sequences used for qPCR

| Gene | Application | Forward primer  （5'→3'） | Reverse primer  （5'→3'） |
| --- | --- | --- | --- |
| Mo-β-actin | RT-qPCR | AGTGTGACGTTGACATCCGT | TGCTAGGAGCCAGAGCAGTA |
| Mo-CTGF | RT-qPCR | TTTGGCCCAGACCCAACTAT | TGGTAACTCGGGTGGAGATG |
| Mo-Fibronectin | RT-qPCR | AAATCGTGCAGCCTCAATCC | GGCTTGCTCTCGCAGTTAAA |
| Mo-CPT1α | RT-qPCR | GGACTCCGCTCGCTCATTC | AGGCAGATCTGTTTGAGGGC |
| Mo-PGC1α | RT-qPCR | ACAACGCGGACAGAATTGAG | GTTTCGTTCGACCTGCGTAA |
| Mo-PPARα | RT-qPCR | TGGTGTTCGCAGCTGTTTTG | AGATACGCCCAAATGCACCA |
| Hu-β-actin | RT-qPCR | CCCTGGAGAAGAGCTACGAG | GGAAGGAAGGCTGGAAGAGT |
| Hu-α-SMA | RT-qPCR | TGTGAAGCAGCTCCAGCTAT | CTTACAGAGCCCAGAGCCAT |
| Hu-FN | RT-qPCR | GGTACAGGGTGACCTACTCG | GGGCTGGCTCTCCATATCAT |
| Hu-Col-I | RT-qPCR | CACCAATCACCTGCGTACAG | GCAGTTCTTGGTCTCGTCAC |
| Hu-CPT1α | RT-qPCR | ATGTCCAGCCAGACGAAGAA | ACTTTCAGGGAGTAGCGCAT |
| Hu-PPARα | RT-qPCR | CACAGCTCACCACCTATGGA | CCCTCAAACAGCTGAGGAGA |
| Hu-PGC1α | RT-qPCR | ATGCAGTGGTCTCAGTACCC | TTGGTGTGAGGAGGGTCATC |
